# Preparation and anti-colon cancer effect of a novel curcumin analogue (CA8): *in vivo* and *in vitro* evaluation

**DOI:** 10.3389/fphar.2024.1464626

**Published:** 2024-11-12

**Authors:** Jie Wen, Lingmao Zhao, Zhuohan Li, Chao Pi, Xianhu Feng, Peng Shi, Hongru Yang, Ligang Chen, Xiaodong Wang, Furong Liu, Yumeng Wei, Ling Zhao

**Affiliations:** ^1^ Key Laboratory of Medical Electrophysiology, Ministry of Education, School of Pharmacy of Southwest Medical University, Luzhou, China; ^2^ Luzhou Key Laboratory of Traditional Chinese Medicine for Chronic Diseases Jointly Built by Sichuan and Chongqing, The Affiliated Traditional Chinese Medicine Hospital, Southwest Medical University, Luzhou, Sichuan, China; ^3^ Central Nervous System Drug Key Laboratory of Sichuan Province, School of Pharmacy, Southwest Medical University, Luzhou, Sichuan, China; ^4^ Luzhou Longmatan District People’s Hospital, Luzhou Third People’s Hospital, Luzhou, Sichuan, China; ^5^ Key Laboratory of Medical Electrophysiology, Ministry of Education, The Affiliated Hospital, Southwest Medical University, Luzhou, Sichuan, China; ^6^ Nanchong Key Laboratory of Individualized Drug Therapy, Department of Pharmacy, Nanchong Central Hospital, Nanchong, Sichuan, China; ^7^ Department of Oncology, The Affiliated Hospital of Southwest Medical University, Luzhou, Sichuan, China; ^8^ Department of Neurosurgery, The Affiliated Hospital, Southwest Medical University, Luzhou, Sichuan, China; ^9^ Department of Hepatobiliary Diseases, The Affiliated Traditional Chinese Medicine Hospital, Southwest Medical University, Luzhou, Sichuan, China; ^10^ Department of Oncology, The Affiliated Traditional Chinese Medicine Hospital, Southwest Medical University, Luzhou, Sichuan, China

**Keywords:** monocarbonyl CU analogues, colon cancer, AKT, JNK, apoptosis

## Abstract

Chemotherapy remains the first choice of treatment for colon cancer despite the inevitable adverse effects. Curcumin (CU) possesses antitumor activity but has poor aqueous solubility, low bioavailability, and weak activity. To address this, nine novel monocarbonyl CU analogues were designed, synthesized, and evaluated in the present study. Among them, CA8 exhibited the highest water solubility, which was approximately 2.37 × 10^6^ times that of CU. In addition, compared with CU, its cytotoxicity on Caco-2 cells (19.2 times/48 h) was stronger. Of note, CA8 arrestedthe cell cycle of Caco-2 cells at the G2/M phase and induced apoptosis. Meanwhile, acute toxicity experiments indicated that KM mice tolerated CA8 for up to 300 mg/kg CA8 (oral administration) and 50 mg/kg CA8 (intraperitoneal injection). The oral administration of CA8 to Sprague Dawley rats exhibited higher AUC (0-t) (6.23-fold) and longer MRT (0-t) (3.35-fold) than that of CU. CA8 also inhibited the proliferation and angiogenesis of tumor cells more than CU and tegafur. Finally, CA8 may exert anti-tumor effects through the activation of JNK pathway and inhibition of AKT pathway. These results suggest that CA8 is a safe and highly effective new drug for colon cancer treatment.

## Graphical Abstract

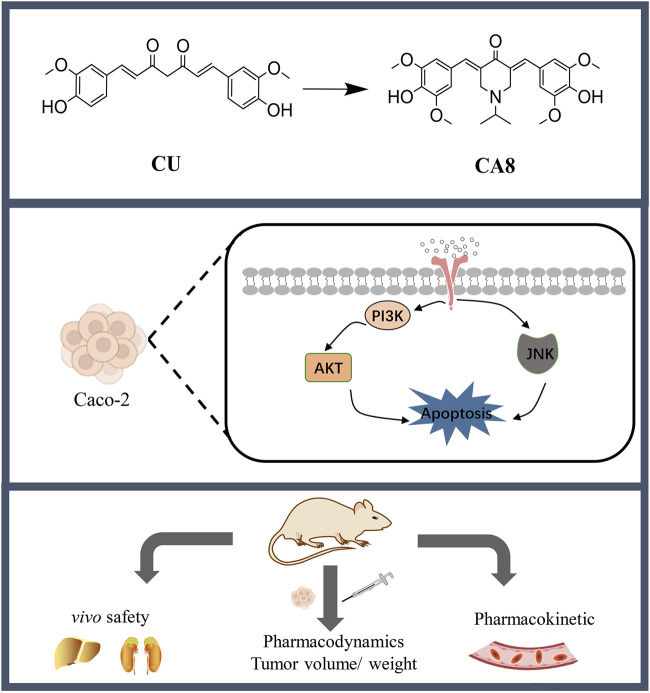

## 1 Introduction

Colon cancer is a common malignant tumor of the digestive tract, being the second leading cause of cancer-related deaths in the world. More than 1.9 million new cases were diagnosed and approximately 935,000 deaths occurred in 2020, with the number of new cases increasing year by year (Sung et al., 2021). Surgical resection is the preferred treatment for stage I - III regional colon cancer. However, colon cancer is prone to relapse or metastasis after surgery (Ye et al., 2019). Numerous studies have confirmed the effective role of adjuvant therapy before and after surgery, and international consensus has been reached on the use of adjuvant treatment in patients with colon cancer at stage I–III. The consensus states that patients in stage I do not require adjuvant chemotherapy and that patients in stage II and III may experience a beneficial effect from adjuvant chemotherapy (Di Costanzo et al., 2003) Although personalized therapy for tumor-specific targets has gradually become a hot topic in the development of anti-colon cancer drugs, drug resistance and high targeted therapy costs lead to a limited use of targeted agents, making classical chemotherapy still the first choice for patients (Schwartzberg et al., 2014; Van Cutsem et al., 2009; Ye et al., 2023). Unfortunately, adverse effects such as myelosuppression, gastrointestinal reactions, and cardiotoxicity caused by standard treatments (such as fluorouracil alone or in combination with oxaliplatin) remain inevitable. Therefore, it is particularly important to find active substances, which are highly toxic against cancer cells and have low side effects, as valid alternatives to chemotherapy for colon cancer.

Natural compounds are an important source of new drugs. CU is one of the main active components of turmeric, a traditional Indian spice that has received extensive attention for its prominent antitumor activity (Martinez-Cifuentes et al., 2015). However, little progress has been made in clinical studies due to its poor aqueous solubility and low bioavailability. Therefore, a structural optimization of CU is necessary to improve its stability, activity, and solubility (Revalde et al., 2015; Ying et al., 2017; Zhao et al., 2019). It has been reported that monocarbonyl CU analogues (MCACs; e.g., EF24 and F35), which are more stable, can be obtained by replacing the unstable diketone structure of CU with a monocarbonyl group, thereby providing CU with a better pharmacokinetic behavior and higher antitumor activity (Schmitt et al., 2017; Wu et al., 2013). However, in-depth studies revealed that they still exhibit some toxicity *in vivo*. Of note, compound 5B, derived from EF24 and F35, was evaluated for antitumor activity only at the cellular level and its efficacy *in vivo* was not assessed (Chen et al., 2018). For enhancing activity, Zhou et al. discovered that pyridine as a distal ring appeared to confer a lower half maximal inhibitory concentration (IC_50_), but the low aqueous solubility remained an obstacle (Zhou et al., 2016). Therefore, most MCACs have shortcomings such as low solubility and low activity, and no studies have evaluated them *in vivo*. Thus, they need to be studied more intensively.

In view of the limitations of the compounds listed above, in this study, MCACs with CU as the lead compound were designed, synthesized, and evaluated with the aim to improve their aqueous solubility, pharmacokinetics, safety, and activity. The compound CA8 showed a significantly enhanced water solubility and strong colon cancer cell toxicity. Therefore, the *in vivo* pharmacokinetic behavior and safety of CA8, as well as its anti-tumor effect and mechanism of action against colon cancer were assessed. These results may provide a theoretical and experimental basis for the search of new drugs to combat colon cancer.

## 2 Materials and methods

### 2.1 Synthesis

The main reagents used in this study were purchased from Aladdin. The target compounds were separated and purified via chromatography silica gel column. The melting point of the target compound was determined using the capillary method, and mass spectrometry analyses were performed using Shimadzu. 1H spectral data were recorded on the 400 MHz spectrometer (Bruker Corporation, Switzerland).

General procedure for the synthesis of CA8 (orange powder, 17.5% yield, melting point 230°C–232°C): First, generate hydrogen chloride gas by heating concentrated sulfuric acid and sodium chloride. Then, slowly introduce the hydrogen chloride gas into stirred glacial acetic acid to ensure it fully dissolves, forming a saturated hydrogen chloride glacial acetic acid solution. Next, appropriate amounts of syringaldehyde (3.47 mmol) and 1-isopropyl-4-piperidone (1.735 mmol) were dissolved in 20 mL of the saturated hydrogen chloride glacial acetic acid solution, and the reaction mixture was stirred at room temperature for 12 h. The mixture was then left to stand for 24 h, filtered, and treated with pure water and absolute ethanol.

The synthesis methods of other compounds and all chemical data are listed in the supplementary information.

### 2.2 Solubility assay

The solubility of the synthesized compounds in water was determined using high performance liquid chromatography (HPLC). First, 1.000 g of each compound was accurately weighed, placed in 100 g of water, and stirred at 25°C for a period of time. Then, the reaction system was allowed to stand for 1 h. The supernatant was quantitatively taken, diluted with methanol to volume, and centrifuged, and the solubility was determined using HPLC.

### 2.3 Antiproliferative activity of MCACs using 3-(4,5-dimethylthiazol-2-yl)-2,5- diphenyltetrazolium bromide (MTT) assay

The human cancer cell lines A549, HepG2, MCF-7, Caco-2, and HeLa and the normal human liver cell line LO2 were all obtained from Shanghai Cell Bank of China. All cells were cultured in RPMI 1640 or DMEM supplemented with 10% fetal bovine serum, 100 U/mL penicillin, and 100 μg/mL streptomycin and incubated at 37°C under 5% CO_2_ (HEPA incubator class100 Thermo company). The medium was replaced three times a week. Cells used in all were in the exponential growth phase.

The cell inhibition rate was evaluated using the MTT assay. Briefly, cells were seeded in a 96-well plate at a density of 3–4 × 10^3^ cells per well and incubated at 37°C for 24 h. The MCACs to be tested were dissolved in dimethyl sulfoxide (DMSO, Merck, Germany) and diluted to five concentrations (3.75, 7.5, 15, 30, 60 μM/L) with RPMI 1640 medium, with each dose analyzed in quadruplicate. The concentration of DMSO was always maintained below 1‰, a concentration that is nontoxic to cells. After incubation for 24 or 48 h, MTT reagent (5 mg/mL) was added to each well and the samples were incubated for 3 h at 37°C in the dark. The absorbance of formazan crystals dissolved in 150 µL of DMSO was measured at 570 nm using a microplate reader to identify the cytotoxic effect of the analogues.

### 2.4 Clonogenic assay

Caco-2 cells were seeded into a 6-well plate at a density of 1 × 10^3^ cells per well and incubated overnight. Then, the medium was replaced, and CA8 was added at the prescribed concentrations (4, 8, or 16 μM/L). After 6 h, the drug-containing medium was replaced with RPMI 1640 medium.

Caco-2 cells in 6-well plates were incubated for 1–2 weeks until the appearance of the colonies, followed by staining with crystal violet and incubation for 1 h at room temperature. After staining, images were captured accordingly.

### 2.5 Hoechst 33,258 staining

Caco-2 cells were seeded in 6-well plates at a density of 5 × 10^5^ cells per well and incubated overnight, followed by the addition of the prescribed concentration (4, 8, or 16 μM/L) of CA8. After 24 h of incubation, cells were fixed and stained with Hoechst 33,258. The morphology of the nucleus was observed with a fluorescence microscope under ×200 magnification.

### 2.6 Apoptosis assay

The apoptotic effect of CA8 was assessed in Caco-2 cells according to the kit instructions. First, Caco-2 cells were treated with CA8 at dose of 4, 8, or 16 μM/L for 24 h. The cells were harvested and suspended in 500 μL annexin-binding buffer. Subsequently, the cells were stained with Annexin V-FITC for 15 min and then incubated with propidium iodide for 5 min at room temperature in the dark. Finally, all samples were evaluated via flow cytometry (FACS Calibur, BD, San Jose, CA, United States). The results obtained were analyzed using the FlowJo software, and the apoptotic rate was calculated.

### 2.7 Cell cycle analysis

Caco-2 cells treated with CA8 at dose of 4, 8, or 16 μM/L for 24 h, they were collected, washed with PBS, and fixed in 70% ethanol overnight. The fixed cells were washed twice with PBS, treated with RNase A (10 mg/mL) and resuspended in 50 μg/mL propidium iodide for staining. Finally, the cell cycle distribution was assessed via flow cytometry (BD Biosciences, CA).

### 2.8 Western blot

The expression of apoptotic proteins and pathways was detected using western blot. Caco-2 cells treated with CA8 at a dose of 4, 8, or 16 μM/L for 24 h were collected and lysed with RIPA lysis buffer for 10 min. The concentration of total proteins was determined using a BCA protein quantitative kit. Total proteins were separated on an SDS-PAGE gel, transferred to PVDF membranes and incubated with 5% skim milk for 2 h. Subsequently, the PVDF membranes were incubated with primary antibodies (AKT 1:2000; Bcl-2 1:2000; Bax 1:5000; caspase-3 1:2,000; caspase-9 1:1000; P-AKT 1:1000; JNK 1:1000; P-JNK 1:1000; IKBα 1:2000; and β-actin 1:100000) at 4°C overnight. The membranes were rinsed three times with TBST and incubated with secondary antibodies (1:5000 dilution) for 2–3 h at room temperature. Finally, proteins were visualized using an ECL chemiluminescent kit (KF001, affinity). In the final step, the ImageJ software was used to analyze the results.

### 2.9 Acute toxicity experiment

All *in vivo* experimental protocols were approved by the animal care committee of the Southwest Medical University and conducted in strict accordance with the laboratory animal care and use of the adopted guidelines. Since among the several compounds, CA8 showed better properties *in vivo* and *in vitro*, its toxicity was evaluated using an acute toxicity test. Twenty-one male KM mice were obtained from the Animal Center of Southwest Medical University and subjected to 1 week of acclimatization before the experiment. Then, the mice were randomly divided into the different groups. CA8 was administered via intraperitoneal injection and oral administration to assess its safety. The oral and injection doses were 5, 50, and 300 mg/kg and 2, 10, and 50 mg/kg, respectively. The body weight of all animals was recorded every day, and their blood samples were collected to analyze relevant biochemical indicators on the 8th day of the experiment. The heart, liver, and kidney tissues of the mice were collected, embedded in paraffin, cut into slices, stained with hematoxylin and eosin (HE), and observed under a light microscope to determine potential pathological changes.

### 2.10 Pharmacokinetics evaluation

After the evaluation of the safety of CA8, 10 male Sprague Dawley (SD) rats were randomly divided into the CA8 and CU groups for the pharmacokinetic test (Ye et al., 2020). The rats were subjected to 1 week of adaptation and were fasted for 6 h before the experiment. CA8 (50 mg kg^−1^) and CU (50 mg kg^−1^) were administered via gavage. Blood samples were collected at 0.03, 0.08, 0.16, 0.25, 0.5, 1, 2, 4, 8 and 12 h and centrifuged at 5,000 rpm for 3 min to obtain the plasma. A mixture of ethyl acetate and methanol was then added to precipitate the proteins and separate the CA8 and CU from the plasma. The samples were then analyzed using HPLC.

### 2.11 *In vivo* anticancer activity of CA8

Twenty 5-week-old male BALB nude mice purchased from SPF (Beijing) Biotechnology Co., Ltd. were used for the *in vivo* pharmacodynamic experiment of CA8. Caco-2 cells (6 × 10^6^ cells in 100 μL PBS) were harvested and subcutaneously injected into the right hip of the mice. Then, they were divided into four groups when the tumor volume reached 100 mm^3^ and treated with saline, CU (100 mg/kg), tegafur (50 mg/kg), or CA8 (100 mg/kg) via gavage every other day. Tumor size and body weight were measured and recorded every other day. At the end of the experiment, the mice were sacrificed and tumors were harvested and weighed. Tumor volumes were calculated by measuring length (L) and width (W) according to the following formula: V = 0.5 × L × W^2^. The tumor tissue was embedded in paraffin, cut into slices, stained with HE, and observed under a light microscope.

### 2.12 Immunohistochemistry

The tumor tissues were dried, fixed for 24 h in 4% paraformaldehyde, embedded in paraffin and cut into sections. The antigen retrieval of the tissue sections was performed in 0.01 M citric acid buffer (pH = 6.0) at 95°C for 3 min. Next, the sections were first incubated with the primary antibody (1:100) anti-CD31 at 4°C overnight and then with the secondary antibody for 2 h at room temperature. Finally, they were treated with 3,3′-diaminobenzidine, and the developed color was observed under a microscope and photographed with a connected digital camera.

### 2.13 Statistical analysis

Statistical analysis was performed using GraphPad Prism 8.0. The results are expressed as the mean ± standard deviation. Tukey’s test was used for inter-group comparison. *P* value of <0.05 was considered statistically significant.

## 3 Results

### 3.1 Design and synthesis

**FIGURE 1:**
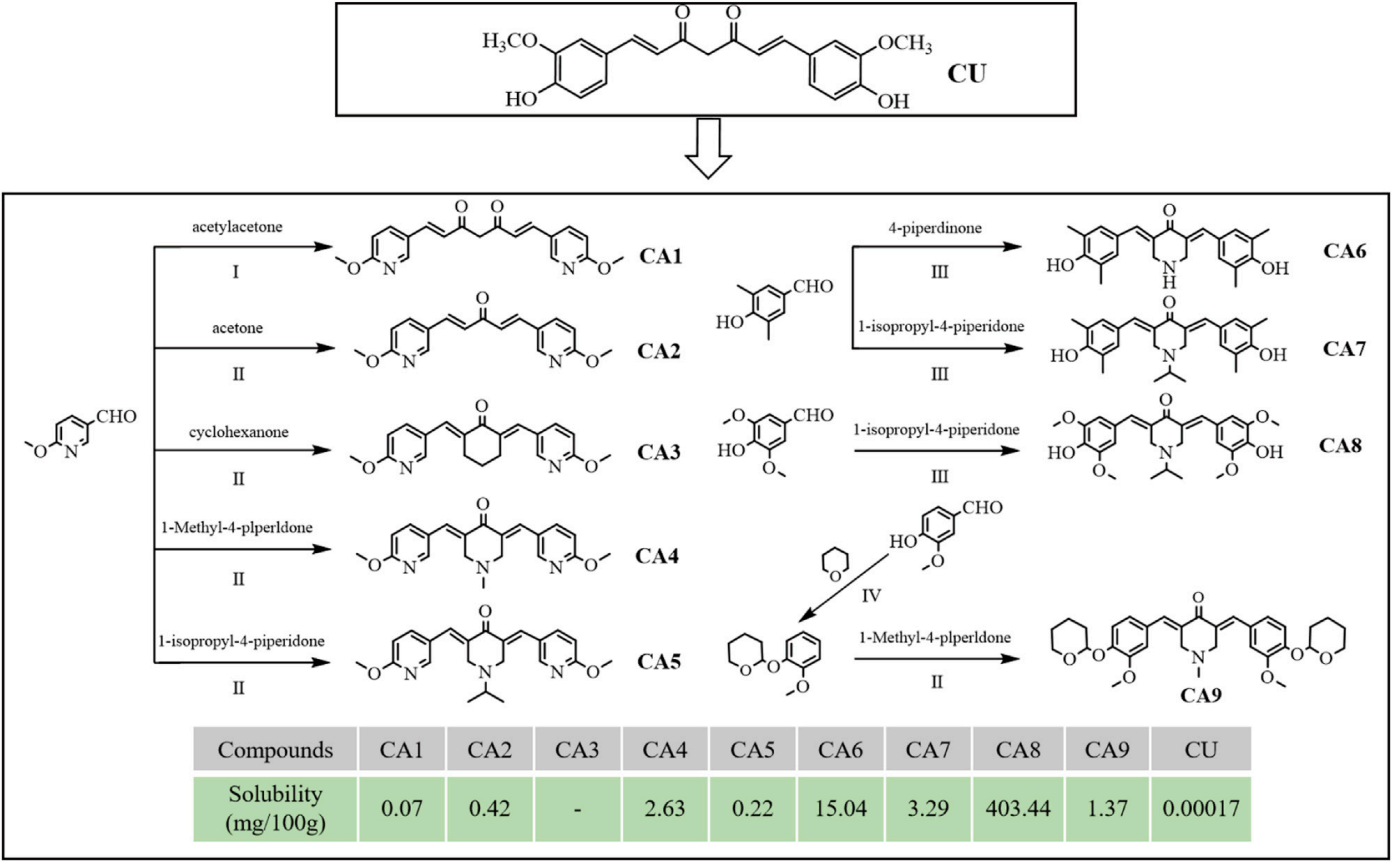
Design, synthesis route, chemical structure and solubility parameters of MCACs. Reagents and conditions: (I) tributyl borate/n-Butylamine/ethyl acetate, Heat to solvent reflux; (II) NaOH/ethyl alcohol, room Temperature; (III) HCl/acetic acid, room temperature; (IV) pyridine-PTSA/dichloromethane, room temperature. Note: Solubility (mg), the mass of solute dissolved when a solid substance reaches saturation in 100 g of solvent at 25°C.

Several MCACs were synthesized using three methods. Compound CA1 was obtained by replacing the phenyl ring of CU with 6-methoxy-3-pyridyl. For the compound CA2, the dicarbonyl group in CA1 was replaced by a monocarbonyl group. For CA4-CA8, a piperidone embedded in two pyridine rings conjugated with flanking C=C bonds was used to replace the central keto-enol curcuminoid moiety. CA3 and CA9 were obtained using similar methods. All synthesis schemes and compound structures are shown in Figure 1. The solubility of all compounds was determined using HPLC, and the results indicated that CA8 exhibited the highest water solubility. All compounds were characterized using Nuclear Magnetic Resonance Hydrogen Spectroscopy (^1^H-NMR) and Liquid Chromatography-Mass Spectrometry (LC-MS). The specific parameters of these compounds such as synthesis method, color, melting point, and LC-MS and ^1^H-NMR spectra are described in detail in the supplemental files.

### 3.2 Antiproliferative activity against human cancer cell lines

**FIGURE 2:**
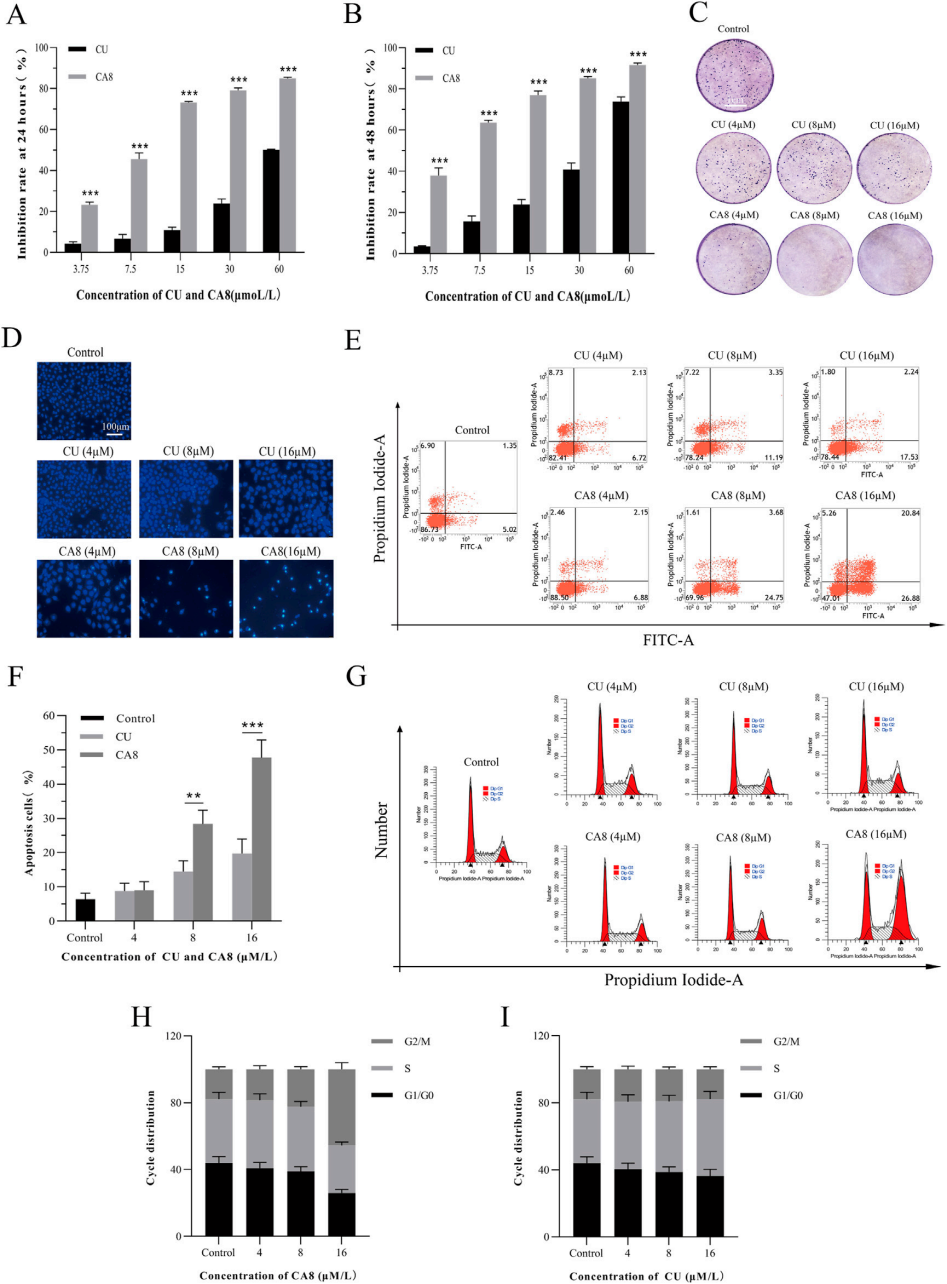
Cytotoxicity, cell apoptosis and cell cycle arrest of CA8 and CU in Caco-2 cells. Inhibitory rate (%) of CU and CA8 on Caco-2 cells at 24 h **(A)**, Inhibitory rate (%) of CU and CA8 on Caco-2 cells at 48 h **(B)**; **(C)** Clonogenic assay of Caco-2 cells treated with increasing concentrations of CA8 and CU. **(D)** Nuclear morphology of Caco-2 cells was assessed by Hoechst 33,258; **(E)** Apoptosis rate calculated by flow cytometry; **(F)** Apoptosis proportion induced by CA8 and CU; **(G)** Cell cycle determination by flow cytometry; **(H**–**I)** The frequency distribution bar graphs of CA8 and CU on G1/G0, S, G2/M phases. **p < 0.01, ***p < 0.001 vs. CA8/CU.

The cytotoxicity of all MCACs was evaluated using the MTT assay on several human cancer cell lines, such as lung cancer cells A549, liver cancer cells HepG2, breast cancer cells MCF-7, cervical cancer cells HeLa and colon cancer cells Caco-2. The results showed that these compounds inhibited the growth of human cancer cells in a dose-dependent manner. Their IC_50_ and corresponding *in vitro* selection coefficients are shown in Table 1. In particular, some MCACs significantly reduced the viability of the five different types of cancer cells after 24 h of treatment compared to CU, except CA1, CA3, and CA6, which showed a low toxicity. CA7 (5.26 ± 0.49 μM/0.87) and CA9 (3.0 ± 0.6 μM/0.70) exhibited a high selectivity index for HeLa and Caco-2 cells, respectively. Our attention focused on CA8 since it showed high water solubility and potent cytotoxicity against Caco-2 cells. CA3 (15.47 ± 0.42 μM/>0.59) and CA6 (9.28 ± 0.68 μM/>0.81) showed significantly enhanced cytotoxicity and high selectivity to liver cancer cells HepG2 after 48 h of treatment. In contrast to the other drugs, CA8 exhibited time dependence and low IC50 (1.71 ± 0.06 μM) in colon cancer Caco-2 cells. The histogram of the inhibition rate of CA8 and CU on Caco-2 cells clearly showed the significant advantages of the new structural compounds (Figures 2A, B). In short, all compounds, except CA1 and CA6, exhibited high cytotoxicity, particularly CA8 and CA7. In view of the influence of the solubility parameters on the bioavailability of drugs *in vivo*, CA8 was used for further experiments to evaluate it as a potential effective candidate compound.

**TABLE 1:** Cytotoxicity and selectivity index (SI) of compounds on a panel of human cancer cell lines and normal cells (24 h and 48 h).

Compound		IC_50_(μM) for cell lines/SI											
		A549		HepG2		MCF-7		HeLa		Caco2		LO2	
24 h	CA1	>60/nc		>60/nc		>60/nc		>60/nc		>60/nc		>60	
	CA2	25.9 ± 1.3/0.07		25.2 ± 1.0/−0.06		21.3 ± 3.1/0.17		18.1 ± 0.8/0.05		15.8 ± 0.3/0.29		31.0 ± 0.41	
	CA3	>60/nc		30.55 ± 5.99/>0.29		>60/nc		29.63 ± 2.57/>0.31		>60/nc		>60	
	CA4	22.9 ± 2.7/−0.54		2.63 ± 0.38/0.37		17.0 ± 1.6/−0.41		4.6 ± 0.5/0.12		7.7 ± 1.8/−0.10		6.11 ± 0.33	
	CA5	27.98 ± 0.77/−0.45		>60/nc		30.10 ± 3.36/−0.49		2.35 ± 0.09/0.62		14.66 ± 0.73/−0.17		9.8 ± 0.51	
	CA6	>60/nc		50.38 ± 15.10/>0.08		>60/nc		>60/nc		>60/nc		>60	
	CA7	36.07 ± 4.76/0.04		22.72 ± 1.37/0.24		54.22 ± 17.34/−0.14		5.26 ± 0.49/0.87		44.44 ± 7.38/−0.05		39.2 ± 1.67	
	CA8	5.66 ± 0.25/−0.12		1.79 ± 1.32/0.38		10.90 ± 4.21/−0.40		4.26 ± 0.52/0.01		4.06 ± 0.89/0.02		4.3 ± 0.21	
	CA9	21.4 ± 2.7/−0.18		11.2 ± 0.5/0.42		22.2 ± 1.8/−0.19		8.4 ± 0.2/0.24		3.0 ± 0.6/0.70		15.0 ± 0.95	
	CU	37.2 ± 2.9/>0.21		43.4 ± 2.9/>0.14		40.0 ± 2.7/>0.18		58.3 ± 2.2/>0.01		>60/nc		>60	
48 h	CA1	28.6 ± 2.2/−0.12		46.9 ± 2.6/0.10		47.3 ± 2.1/0.17		56.5 ± 3.7/-0.01		>60/nc		57.6 ± 4.83	
	CA2	16.1 ± 0.8/0.04		19.8 ± 0.4/−0.06		16.8 ± 0.6/0.03		18.1 ± 0.8/−0.05		10.4 ± 0.7/0.23		17.5 ± 1.09	
	CA3	>60/nc		15.47 ± 0.42/>0.59		>60/nc		38.27 ± 1.11>0.2		47.44 ± 0.91/>0.1		>60	
	CA4	12.5 ± 0.2/−0.72		1.95 ± 0.20/0.10		7.8 ± 0.7/−0.47		2.7 ± 0.5/−0.03		9.4 ± 1.0/−0.59		2.5 ± 0.13	
	CA5	34.71 ± 1.89/−0.7		2.18 ± 0.40/0.50		4.94 ± 1.42/0.14		1.81 ± 0.36/0.58		4.87 ± 0.19/0.15		6.9 ± 0.46	
	CA6	>60/nc		9.28 ± 0.68/>0.81		>60/nc		>60/nc		>60/nc		>60	
	CA7	43.84 ± 5.13/−0.72		2.71 ± 0.49/0.49		18.24 ± 0.52/−0.34		1.26 ± 0.18/0.82		7.44 ± 0.49/0.05		8.4 ± 0.41	
	CA8	9.31 ± 1.08/−0.66		1.84 ± 0.33/0.04		7.23 ± 1.55/−0.55		1.96 ± 0.09/0.01		1.71 ± 0.06/0.07		2.0 ± 0.04	
	CA9	11.7 ± 0.5/−0.37		10.7 ± 1.6/−0.24		10.9 ± 1.3/−0.31		4.3 ± 0.1/0.07		2.09 ± 0.32/0.38		5.0 ± 0.09	
	CU	34.4 ± 1.5/−0.09		27.4 ± 0.3/−0.01		22.3 ± 1.6/0.10		17.3 ± 0.8/0.20		33.0 ± 2.1/−0.08		27.6 ± 1.36	

Abbreviations: IC_50_, concentrations of drugs needed to kill half the number of cancer cells; SI, the logarithm of the ratio of the IC_50_ value of normal cells (LO2) to the IC_50_ value of cancer cells (A549, HepG2, MCF-7, HeLa, and Caco-2); nc, not calculated.

The plate cloning experiment was performed to confirm the cytotoxicity of compound CA8 on Caco-2. Indeed, compared with the control, the treatment of Caco-2 with CA8 dissolved in the medium showed Caco-2 colony formation inhibition (Figure 2C). However, the treatment with CU at three different concentrations did not have a significant inhibitory effect on the colony formation of Caco-2 cells. Therefore, the MTT results mentioned above were validated using the plate cloning assay.

### 3.3 Effect of CA8 on cell apoptosis and cell cycle analysis

Flow cytometry was used to analyze the effect of CA8 on Caco-2 cell cycle distribution and apoptosis to investigate its potential molecular mechanism on the growth inhibition of tumor cells. Both CU and CA8 induced early and late apoptosis in a concentration-dependent manner, but CA8 induced more apoptosis than CU at high and medium concentrations (Figures 2E, F), and this difference was statistically significant (*P* < 0.01; *P* < 0.001). The results of the qualitative experiment (Figure 2D) were basically consistent with those of the quantitative experiments above using the same doses. In addition, the periodic analysis showed that compound CA8 induced G2/M phase arrest in a concentration-dependent manner, and the S phase arrest at high concentration was significantly smaller than that at G2/M phase (Figures 2G, H). In contrast, CU induced S-phase arrest only to a lesser extent (Figure 2I).

### 3.4 Mechanism of action of CA8

**FIGURE 3:**
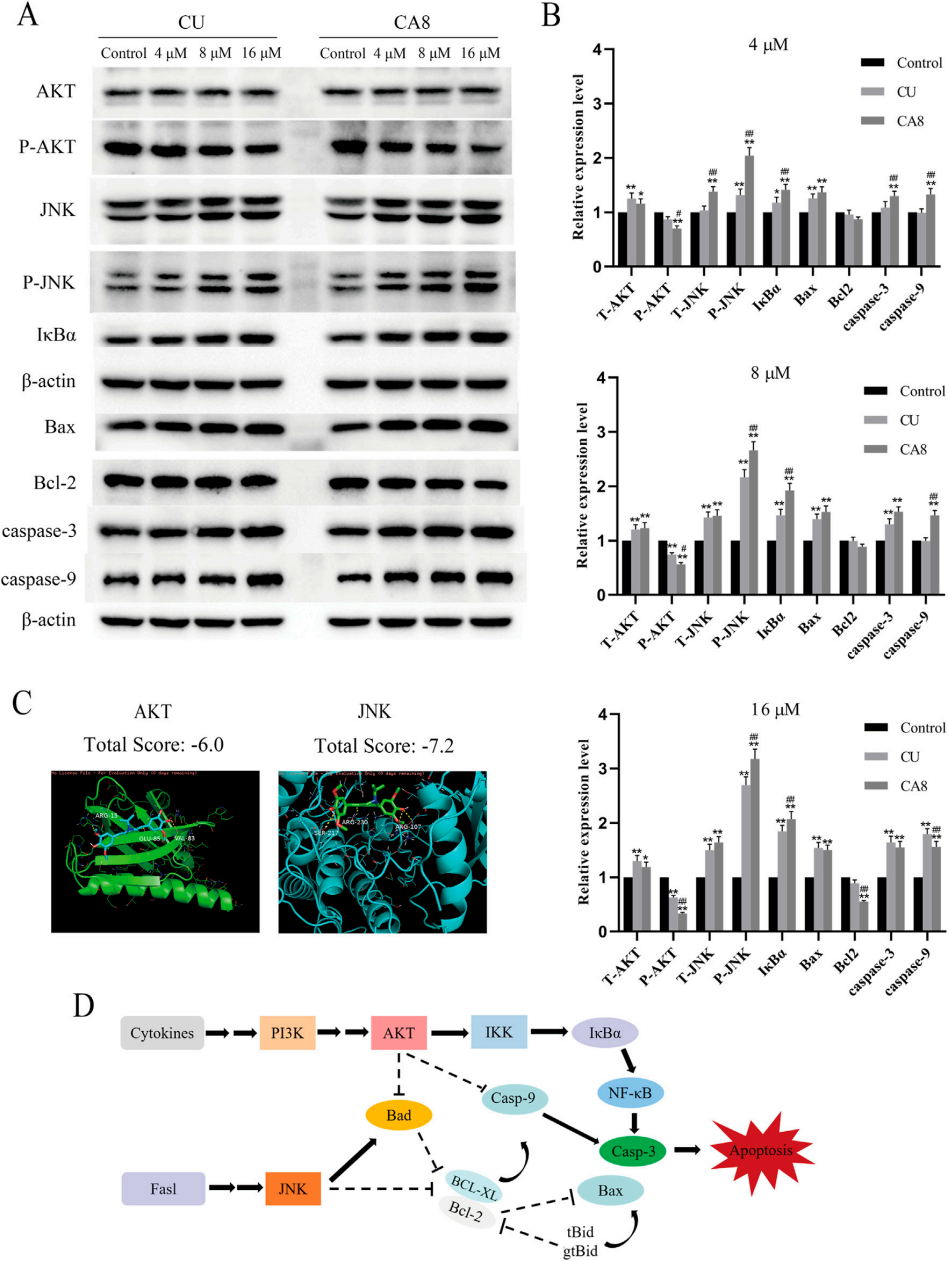
AKT and JNK signaling pathways are involved in CA8-induced colon cancer cell death. **(A)** Caco-2 cells were treated with CA8 (4 μM, 8 μM, 16 μM) for 24 h, the protein levels of AKT, P-AKT, JNK, P-JNK, IκBα, Bax, Bcl-2, caspase-9 and caspase-3 were determined by Western blot; **(B)** The relative level expression of each protein at three treatment concentrations of 4 μM, 8 μM, and 16 μM (n = 3); **(C)** The molecular docking results of CA8 with AKT and JNK; **(D)** The relationship between signaling pathways that CA8 may be involved in. *p < 0.05, **p < 0.01 vs. CA8 and CU/Control. #*p* < 0.05, ##*p* < 0.01 vs. CA8/CU.

The molecular docking technology was used to explore the mechanism of the antitumor effect of CA8 by the induction of apoptosis, predicting that CA8 binds the AKT and JNK protein with a high affinity (total score ≤ −6) (Figure 3C). The relative expression of AKT, JNK, and NF-κB signaling pathways and apoptosis-related proteins such as Bax, Bcl-2, caspase-9 and caspase-3 involved in them were measured using western blot (Figure 3A). The anti-tumor effect of CA8 was confirmed by comparing its effect with that of CU. The results showed that CA8 and CU induced the expression of P-JNK and IκBα in a concentration-dependent manner (*P* < 0.01) and inhibited the expression of P-AKT (*P* < 0.01) compared to the control; CA8 showed a stronger effect than CU at each concentration (*P* < 0.05) (Figure 3B). The expression of the pro-apoptotic protein Bax, caspase-3, and caspase-9 increased in a concentration-dependent manner after treatment with CA8, with a better effect at low and medium concentrations (*P* < 0.01). In addition, the anti-apoptotic protein BCL-2 was significantly inhibited by CA8 at high concentration, and this result was statistically significant compared with that in the control group and CU group (*P* < 0.01). The connections between the above possible signal paths are illustrated in Figure 3D.

### 3.5 *In vivo* acute toxicity of CA8

**FIGURE 4:**
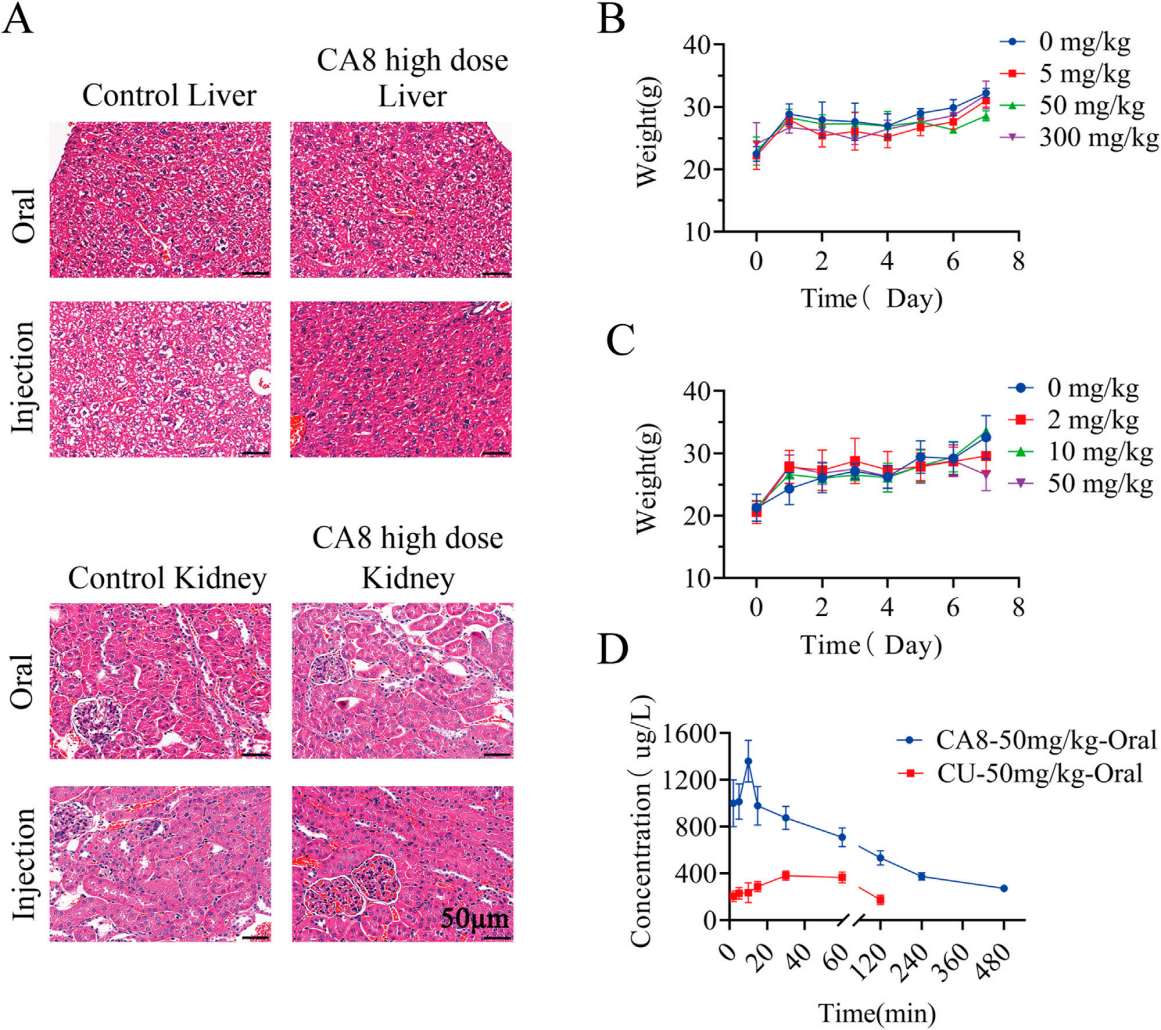
The effect of oral or injectable compound CA8 on safety in KM mice. **(A)** Histopathological sections of liver and kidney of mice in high dose group and control group; **(B)** The changes in body weight of mice after oral administration; **(C)** The changes in body weight of mice after intraperitoneal injection; Data are mean ± SD (n = 3). **(D)** Plasma drug concentrations vs. time profiles following oral administration of CA8 or CU; Data are mean ± SD (n = 5).

The acute toxicity of CA8 was evaluated by treating mice with single oral and intraperitoneal injection. No morbidities and deaths occurred during the whole experimental period, and no abnormal behavior of mice was observed in the high dose group. The weight change curve of the mice during the experiment was recorded and plotted, partially reflecting the safety of CA8 (Figures 4B, C). The blood biochemistry analysis revealed that there was no significant difference in the blood parameters ALT, AST, ALP, CREA, UA, and UREA between the experimental group and control group (Table 2). In addition, the sections of the liver and kidney showed that the morphology of the cells was normal, and no significant pathological damage due to the tested drugs was observed in the experimental animals after oral and intravenous treatment at different doses (Figure 4A). Therefore, the above results confirmed that CA8 was well tolerated by mice and was safe to some extent.

**TABLE 2:** Effect of CA8 as a single acute oral dose at 5, 50 and 300 mg/kg body weight and single acute injection dose at 2, 10 and 50 mg/kg body weight on serum biochemical parameters in KM mice (Mean ± SE; n = 3).

Parameters	Effect of CA8 in various parameters as a single oral/injection dose in mice				
		Control	5 (2) mg/kg	50 (10) mg/kg	300 (50) mg/kg
oral	ALT	51.7 ± 14.62	49.70 ± 5.25	61.30 ± 1.30	44.63 ± 11.95
	AST	134.70 ± 15.32	209.40 ± 69.21	269.23 ± 67.81	277.57 ± 160.53
	ALP	239.27 ± 106.93	208.57 ± 19.55	299.77 ± 72.56	266.97 ± 26.38
	CREA	14.73 ± 0.83	10.34 ± 0.87	12.50 ± 2.78	12.90 ± 1.61
	UA	104.30 ± 44.61	72.27 ± 18.54	124.17 ± 19.83	128.03 ± 33.61
	UREA	8.49 ± 0.54	6.70 ± 0.12	6.53 ± 0.65	7.80 ± 1.27
injection	ALT	55.73 ± 4.45	59.77 ± 3.00	54.87 ± 3.50	48.27 ± 2.77
	AST	122.43 ± 36.0	257.50 ± 114.60	100.13 ± 28.26	141.83 ± 50.47
	ALP	192.33 ± 18.0	220.33 ± 47.16	175.93 ± 95.23	226.47 ± 65.98
	CREA	10.03 ± 1.20	17.13 ± 3.30	11.60 ± 1.60	12.13 ± 1.12
	UA	66.27 ± 18.02	171.93 ± 1.10	47.57 ± 14.59	55.77 ± 13.47
	UREA	7.75 ± 0.50	6.84 ± 0.29	6.84 ± 0.94	9.19 ± 1.02

### 3.6 Pharmacokinetic evaluation of CA8

The pharmacokinetic behaviors of CA8 and CU in SD rats were delineated via a concentration-time curve (Figure 4D) to evaluate the oral bioavailability of CA8. CU was completely cleared after 2 h while CA8 was still detected after 8 h. At all sampling points, the plasma concentrations of CA8 were significantly higher than those of CU. The pharmacokinetic parameters are shown in Table 3. CA8 exhibited higher bioavailability (6.23-fold), mean residence time (3.35-fold), and biological half-life (2.38-fold) than CU.

**TABLE 3:** Pharmacokinetic parameters in rats following oral administration of CA8 and CU.

Parameter	Unit	CA8	CU
AUC_(0-t)_	μg/L*min	222,562.75	35,697.63
MRT_(0-t)_	min	181.06	54.03
t_1/2z_	min	178.68	74.82
T_max_	min	10.00	30.00
C_max_	μg/L	1,359.75	380.00
CL_z_	L/min/kg	0.19	0.90
V_z_	L/kg	49.53	97.58

These parameters confirmed that CA8 possessed better pharmacokinetic behaviors than CU, providing a basis for a potentially improved drug efficacy.

### 3.7 *In vivo* antitumor effect of CA8

**FIGURE 5:**
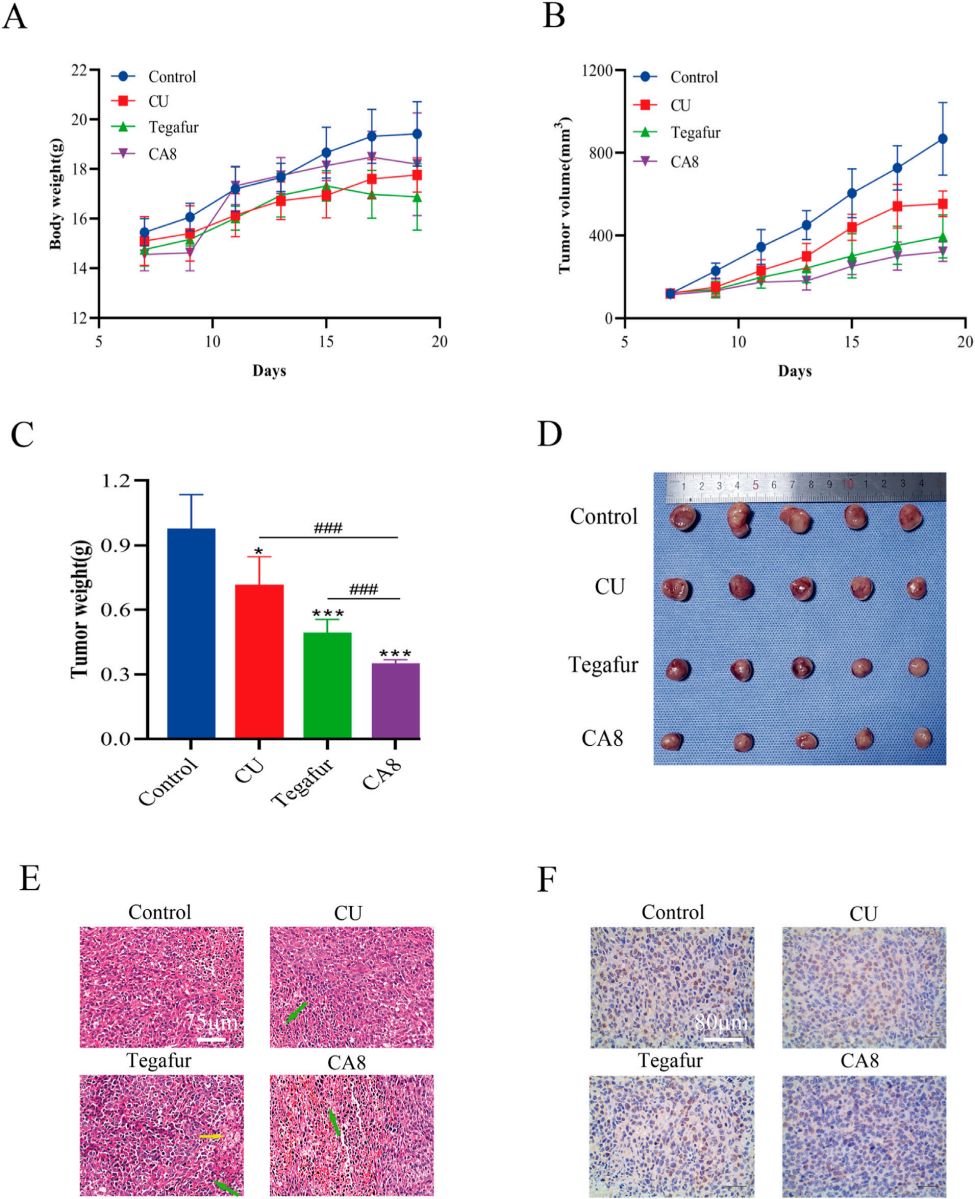
CA8 inhibits growth of Caco-2 xenograft tumors *in vivo*. **(A)** Body weight changes in nude mice during the experiment; **(B)** Tumor volume changes in nude mice during the experiment; **(C)** The final weight of the tumors in each group; **(D)** The final size of the tumors in each group; **(E)** The results of tumor mass HE staining in groups (green arrow: necrotic tumor cells, yellow arrow: foam cell); **(F)** The expression of ki67 in tumor tissues was determined by immunohistochemistry. Data are mean ± SD (n = 5); *p < 0.05, ***p < 0.001 vs. CA8, Tegafur and CU/Control. ###*p* < 0.001 vs. CU and Tegafur/ CA8.

Based on the above *in vitro* experimental results, the anti-tumor effect of CA8 was evaluated *in vivo*. The weight of mice after 13 days of therapy increased to varying degrees. The change trend in the body weight of the mice in the CA8 group was basically consistent with that in the control group, suggesting that it did not lead to a significant toxicity (Figure 5A). CA8 exerted a more significant inhibitory effect on tumor growth than CU and tegafur (Figures 5B–D; *P* < 0.05, *P* < 0.001, *P* < 0.001) compared with the control group. Specifically, the anti-tumor effect was CA8 > tegafur > CU, and the difference was statistically significant (*P* < 0.01). The tumor inhibition rate of CA8 was 63.93%. HE staining results indicated that the tumors in the control group remarkably grew and had abundant microvessels, while the CA8 group showed a large necrotic area in the tumor mass (Figure 5E). The immunohistochemical results revealed that CA8 significantly reduced the expression of Ki67 in tumor tissues (Figure 5F). These findings further confirmed that CA8 significantly inhibited the growth of blood vessels in the tumor, consequently inhibiting tumor growth. Taken together, CA8 inhibited tumor growth more effectively than the other tested drugs, which was consistent with the results of *in vitro* pharmacodynamics.

## 4 Discussion

Active ingredients from natural plants have emerged as a treasure-house for the development of new antitumor drugs (Guo et al., 2020). CU is an active monomer extracted from plants with anti-colon cancer activity *in vivo* and *in vitro* (Selvam et al., 2019). However, its poor aqueous solubility and activity remain major challenges for its clinical application. Hence, several novel MCACs with antitumor activity were designed and synthesized in this study. Compared with CU and CA1, all MCACs (CA2-CA9) showed significant toxicity except for CA6. These results are similar to those reported by Min et al. (2020), Pan et al. (2016), Qu et al. (2013), Zhao et al. (2013). When pyridine was used as a distal ring, MCACs with piperidone ring linkers (CA4 and CA5) exerted a higher cytotoxicity. Interestingly, 3, 5-dimethyl-4-phenol exhibited high antitumor selectivity when used as a distal ring, such as in the case of CA6 on HepG2 cells and CA7 on HeLa cells. Although CA9 exhibited high selectivity, its use as a cancer drug was constrained by its instability. CU is almost insoluble in water (Dey and Sreenivasan, 2014; Ma et al., 2019; Nelson et al., 2017). Al-Hujaily et al. found that the solubility of a novel CU analogue (PAC) in water was 27 times that of CU (Al-Hujaily et al., 2011). Among all compounds, CA8 showed the highest water solubility, i.e., 2.37 × 10^6^ times that of CU. The distal ring structure of CA8, which may be extrapolated from the structural features of PAC, may be the reason for the improved water solubility. Moreover, CA8 showed a strong colon cancer toxicity, and our speculation was that CA8 has the best oral bioavailability due to its high solubility. In addition, the steric hindrance of the two methoxy groups to the phenolic hydroxyl group possibly enhances the stability of CA8. These assumptions are reasonable based on the present study results and previous reports.

Cell apoptosis and cell cycle are important phenomena involved in cell growth and proliferation. The induction of cancer cell cycle arrest or apoptosis through multiple mechanisms is a strategy to control cancer cell proliferation (Patel et al., 2020). The results revealed that CA8 was significantly stronger than CU in arresting Caco-2 cell cycle and inducing apoptosis. Moreover, many apoptotic cells were observed and dense granular lumpy fluorescence was observed in the apoptotic cell nucleus in the CA8 group. All cancer cells used in this study were highly sensitive to CA8, which indirectly proved that CA8 has broad-spectrum anti-tumor properties. In view of this effect, our speculation was that CA8 is a multitarget drug. AKT and JNK proteins bind to CA8 with high affinity (Figure 3C), which served as a foundation for the western blot experiments. Through Western blot analysis, we found that CA8 may promote tumor cell apoptosis and inhibit proliferation by regulating the AKT, NF-κB, and mitochondrial apoptosis pathways. Specifically, we observed that CA8 significantly suppresses the phosphorylation level of AKT. The reduction in AKT phosphorylation has been demonstrated to exert a marked inhibitory effect on tumor proliferation (He et al., 2021; Vanhaesebroeck et al., 2010). As a downstream transcription factor of the AKT signaling pathway, NF-κB antagonizes apoptosis by inducing the transcription of anti-apoptotic genes, while IκBα inhibits the expression of NF-κB (Guo et al., 2024; Liu et al., 2020). In our study, IκBα was significantly upregulated following CA8 treatment, suggesting that the apoptotic effects of CA8 on tumor cells may also involve the NF-κB signaling pathway. Additionally, JNK activation leads to apoptosis, and phosphorylated JNK (P-JNK) can directly phosphorylate Bcl-2, indirectly activating Bax (Chen et al., 2018; Chen et al., 2016; Putcha et al., 2003). A low B-2/Bax ratio triggers apoptosis (Pihán et al., 2017). In this study, we found that CA8 increased the phosphorylation level of JNK in a dose-dependent manner and decreased the Bcl-2/Bax ratio, revealing the involvement of the JNK signaling pathway in mediating CA8’s antitumor activity. Lastly, we also observed that CA8 significantly upregulated the protein levels of caspase-9 and caspase-3, which are known to promote mitochondrial apoptosis (Kim et al., 2015; Li et al., 2017). In summary, CA8 may exert its antitumor effects by inhibiting AKT phosphorylation, suppressing the NF-κB signaling pathway, and activating the JNK-mediated mitochondrial apoptosis pathway.

Appropriate pharmacokinetic characteristics are the key factors affecting drug accumulation *in vivo* (Aggarwal and Sung, 2009). CU has poor solubility and consequently poor bioavailability (Li et al., 2014). Solubility may be improved through structural modification, one of the important optimization strategies for lead compounds. The solubility of CA8 was greatly improved in this study, leading to significant changes in its pharmacokinetic parameters. Subsequently, an animal model of xenograft nude mice bearing Caco-2 cells was developed to determine the *in vivo* antitumor effects of CA8 and CU. CA8 exerted a stronger inhibitory effect on tumors than CU that was almost consistent with the results of the *in vitro* experiments, which may be attributed to its higher bioavailability via oral administration. In addition, its efficacy was significantly better than that of the clinical drug tegafur.

Drug safety is an important parameter for the evaluation of the efficacy of anticancer drugs. Acute toxicity is used to assess the ability of a substance to induce adverse reactions within a short period of time after treatment. The preliminary acute toxicity experiments performed in this study showed that CA8 did not exert any evident toxicity. Moreover, the weight gain curve of the CA8 group was almost consistent with that of the control group during 2 weeks of continuous body weight monitoring. This result indicated that the toxicity of CA8 was lower than that of tegafur, indirectly suggesting no evident toxicity during treatment and validating the results of the acute toxicity experiments.

In conclusion, several MCACs with broad-spectrum antitumor activity were successfully designed and synthesized. CA8 showed superior chemical and physical qualities and excellent safety, as well as an anti-colon cancer activity *in vitro* and *in vivo* by activating the JNK pathway and inhibiting the AKT pathway. Therefore, CA8 is a potential promising novel anti-colon cancer drug, which deserves further investigation.

## Data Availability

The original contributions presented in the study are included in the article/supplementary material, further inquiries can be directed to the corresponding authors.
